# Automated Posterior Scleral Topography Assessment for Enhanced Staphyloma Visualization and Quantification With Improved Maculopathy Correlation

**DOI:** 10.1167/tvst.13.10.41

**Published:** 2024-10-30

**Authors:** Ying Xiang Han, Xiao Xiao Guo, Ya Xing Wang, Jost B. Jonas, Xi Chen, Xiao Fei Wang

**Affiliations:** 1 Laboratory for Biomechanics and Mechanobiology of Ministry of Education, Beijing Advanced Innovation Center for Biomedical Engineering, School of Biological Science and Medical Engineering, Beihang University, Beijing, China; 2 Department of Ophthalmology, Beijing Anzhen Hospital, Capital Medical University, Beijing, China; 3 Beijing Institute of Ophthalmology, Beijing Tongren Hospital, Capital Medical University, Beijing Ophthalmology and Visual Sciences Key Laboratory, Beijing, China; 4 Rothschild Foundation Hospital, Institut Français de Myopie, Paris, France; 5 Department of Ophthalmology, Beijing Friendship Hospital, Capital Medical University, Beijing, China; 6 Eye Hospital, School of Ophthalmology and Optometry and School of Biomedical Engineering, Wenzhou Medical University, Wenzhou, China

**Keywords:** high myopia, posterior staphyloma, mri, deep learning, myopic traction maculopathy

## Abstract

**Purpose:**

To quantitatively characterize the posterior morphology of high myopia eyes with posterior staphyloma.

**Methods:**

Surface points of the eyeball were automatically extracted from magnetic resonance imaging scans using deep learning. Subsequently, the topography of posterior staphylomas was constructed to facilitate accurate visualization and quantification of their location and severity. In the three-dimensional Cartesian coordinate system established with surface points, measurements of distances (D) from each point to the hypothetical pre-elongation eye center within the eyeball and local curvatures (C) at each point on the posterior sclera were computed. Using this data, specific parameters were formulated. The concordance of these parameters with traditional staphyloma classification methods and their association with myopic traction maculopathy (MTM) grades based on the ATN classifications were investigated.

**Results:**

The study included 102 eyes from 52 participants. The measured parameters, particularly the variance of distance (D_var_) and the maximum value of the curvature and distance product (C · D_max_), demonstrated efficacy in differentiating various types of posterior staphyloma and exhibited strong correlations with the grades of MTM.

**Conclusions:**

The automated generation of the posterior scleral topography facilitated visualization and quantification of staphyloma location and severity. Simple geometric parameters can quantify staphyloma shape and correlate well with retinal complications. Future works on expanding these measures to more imaging modalities could improve their clinical use and deepen insights into the link between posterior staphyloma and related retinal diseases.

**Translational Relevance:**

This work has the potential to be translated into clinical practice, allowing for the accurate assessment of staphyloma severity and ultimately improving disease management.

## Introduction

Posterior staphyloma is an outpouching or bulging of the posterior sclera of the eyeball,^1^^–^^3^ commonly associated with high myopia and pathologic myopia.^4^^–^^8^ It is markedly associated with myopic maculopathy.^9^^–^^12^ A valid classification of posterior staphylomas plays a crucial role in the management and research of pathologic myopia.^13^^–^^16^

Previous studies have primarily graded posterior staphylomas using ophthalmoscopy and conventional photographic fundus images. Later, three-dimensional (3D) imaging techniques, such as magnetic resonance imaging (MRI) and optical coherence tomography (OCT), have been applied. The 3D MRI, in particular, has significantly enhanced the visualization of staphylomas assessing the whole eye shape without optical distortions commonly associated with OCT imaging.^17^^–^^20^ Despite these advancements, most studies have relied on subjective classification methods, merely categorizing the posterior scleral shape based on its appearance. For instance, Moriyama et al.^19^ classified the posterior shape of eyes into four types based on ocular elongation and symmetry: nasally distorted, temporally distorted, cylinder-shaped, and barrel-shaped. Guo et al.^14^ and Luo et al.^16^ expanded this classification, introducing additional staphyloma types such as spheroidal, conical, ellipsoidal, and barrel-shaped staphylomas. Although these studies have broadened our understanding of the posterior eye shape, they have predominantly lacked quantitative measures, relying on subjective assessments with considerable variability. Recent methodologies, such as the application of Zernike coefficients for the characterization of the eyeball morphology have attempted to bridge this gap.^21^ However, the explainability has been limited, and their correlations with macular complications have remained unclear. Therefore a more precise, quantitative measurement of the posterior scleral shape is needed for the classification of posterior staphylomas with clinical relevance.

The aim of this study was to develop methods to automatically generate a posterior scleral topography from 3D MRI images and to establish quantitative measurements for staphyloma shape. The topography and quantitative measurements were designed to improve the conventional classification of posterior staphylomas and to investigate its association with the severity of macular complications. Ultimately, this research intends to provide a more objective and consistent methodology for posterior scleral shape assessment, potentially leading to improved diagnostic and treatment strategies for conditions associated with posterior staphyloma.

## Methods

### Subjects Recruitment and MRI Imaging

The hospital-based study included highly myopic patients who participated in a follow-up study on high myopia. All participants had an age ranging between 20 and 80 years and had bilateral high myopia, defined as an axial length (AL) of ≥26.5 mm or a refractive error of ≤−6 diopters (D) (spherical equivalent), without secondary causes of myopia, any history of refractive or intraocular surgery, or any systemic conditions. This study was approved by the Medical Ethics Committee of the Beijing Friendship Hospital and adhered to the tenets of the Declaration of Helsinki.

MRI imaging of the orbital region was performed using a 3T MR scanner (Discovery MR750; GE Medical Systems, Chicago, IL, USA) with an eight-channel head coil. The 3D T2-weighted MRI volumes with an in-plane resolution of approximately 0.5 mm and a slice thickness of 1 mm were acquired with parameters of TR = 2500 ms, TE = 248 ms, and flip angle = 90°. Individuals were positioned comfortably in a supine position with their eyes closed and were instructed to minimize eye movements during image acquisition. The MRI image acquisition time for the sequence used in this study averaged 3.38 ± 0.57 minutes. The MRI system includes built-in correction mechanisms for minor motion artifacts. Additionally, a manual review of the MRI image quality was performed. If the scans showed blurry eyeball boundaries, misalignment of tissue structures between slices, or other motion artifacts, the scan was retaken. This process ensured that only high-quality images were included in our analysis.

### Automated Segmentation of the Eyeball Using Deep Learning

A deep learning model was developed to automatically segment the eyeball from the surrounding orbital tissue on the MRI images. To develop the model, we first manually segmented 570 two-dimensional MRI images using 3D Slicer (https://www.slicer.org/) in the sagittal plane to create a ground truth dataset for training and validation. Manual segmentation was primarily performed in the sagittal plane with necessary corrections using information from coronal or axial views of the 3D volume images to ensure accuracy. Once trained, the model was used to delineate the globes from the orbital tissue in all MRI volumes automatically.

A custom-designed U-shaped convolutional neural network (U-net) was employed, with its architecture detailed in the Supplementary material. The U-net consisted of feature blocks, each comprising a layer normalization, a depth-wise convolution (kernel size of 7 and stride of 1), another layer normalization, and a multilayer perceptron. Skip connections connected the input and output of each feature block. The input was divided into nonoverlapping patches using a convolution (kernel size and stride of 4), followed by three stages of down-sampling and up-sampling. Skip connections concatenated the outputs of different stages. The network utilized the Adam optimizer with a learning rate of 0.001 and a cross-entropy loss function. The Dice coefficient, ranging from 0 to 1, measured the segmentation accuracy, with values above 0.9 indicating excellent overlap between automated and manual segmentations.

### 3D Reconstruction and Posterior Scleral Topography

**Figure 1.**
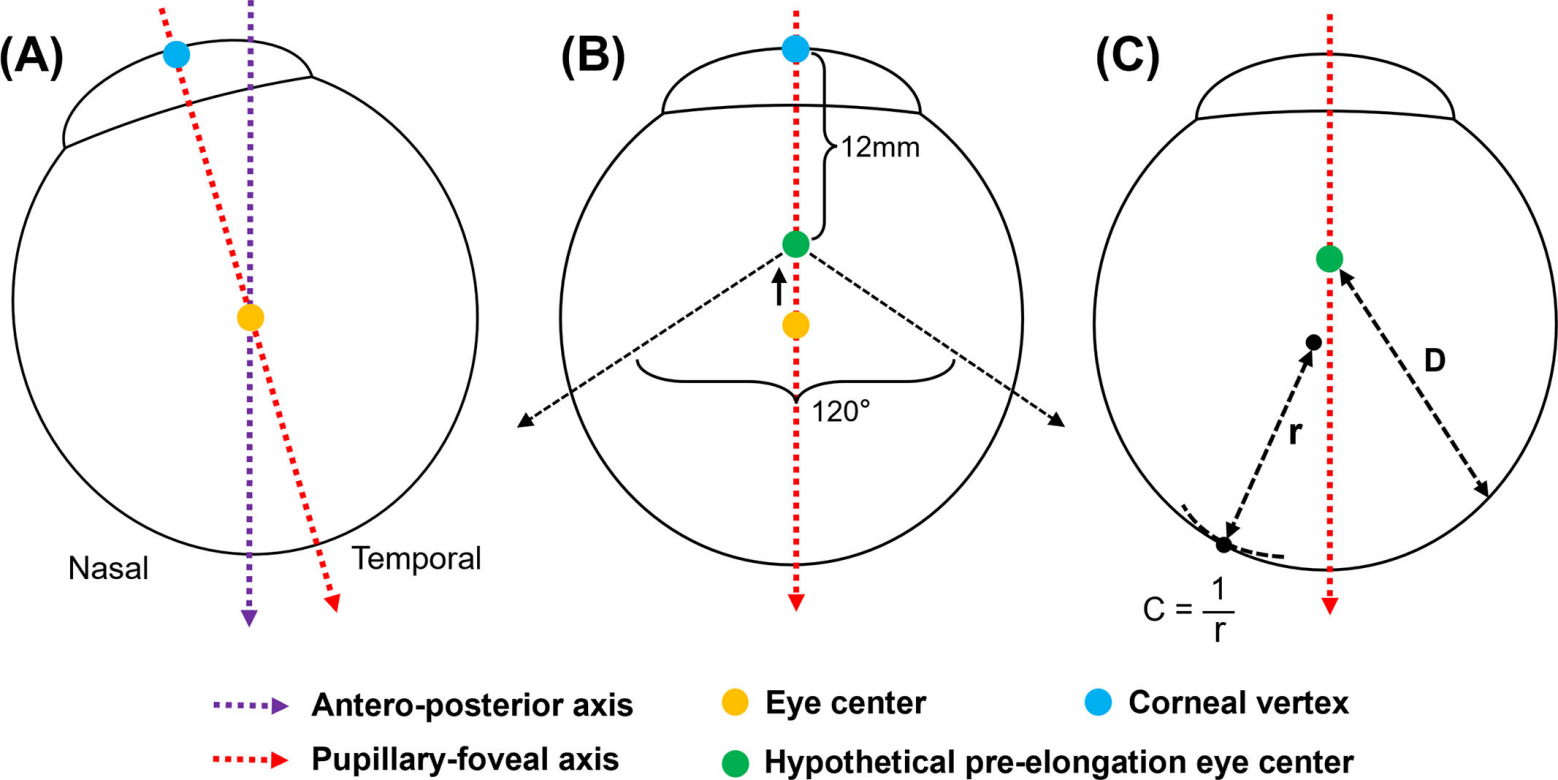
Parameter analysis process of the posterior surface of the eyeball. (**A**) The eyeball center (*yellow dot*) and the corneal vertex (*blue dot*) were obtained from a maximally inscribed sphere within the eyeball surface and the point most distant to this center on the anterior surface, respectively. The line connecting these two points was defined as the pupillary-foveal axis. (**B**) The origin of the coordinate system was set at 12 mm posterior to the corneal vertex, approximating the center of a normal eyeball with an axial length of about 24 mm. Parameters were measured and the posterior scleral topography was generated for a posterior region spanning 120°. (**C**) Distance (*D*) from each point to the hypothetical pre-elongation eye center (*green dot*) and the curvature (*C*) based on fitting a small spherical surface to the local points within a 3 mm radius region centered at each point, were measured over a 120° range of the posterior eyeball.

Before the automatic segmentation of the eyeball, the MRI images were resampled in the sagittal direction to achieve a slice spacing of 0.5 mm, resulting in a uniform pixel spacing of 0.5 mm in all three dimensions. After the segmentation, Gaussian smoothing and the marching cubes algorithm were applied to the segmented eyeball to obtain the point cloud of the eyeball surface. A spatial Cartesian coordinate system based on the MRI volume was established for these points: the naso-temporal direction as the X-axis, the antero-posterior as the Y-axis, and the infero-superior direction as the Z-axis. Because eyes were closed during imaging, maintaining a primary gaze position was exactly not possible. Therefore, this initial coordinate system was refined to align the Y-axis with the antero-posterior direction of the eyeball, i.e., the pupillary-foveal axis. This refinement involved identifying a maximally inscribed sphere within the eyeball surface using a search algorithm. The center of this sphere was considered to be on the pupillary-foveal axis. We identified the point on the anterior sphere surface most distant from the sphere center, and considered it to be the corneal vertex. The line connecting the corneal vertex and the sphere center defined the pupillary-foveal axis (Fig. 1A). This method was validated through manual examination of the axes on the MRI images and compared against the method proposed by Hoang et al.^22^ We found strong agreement between these two methods (see Supplementary Material for details). It is worth noting that instead of directly fitting the entire eyeball surface, we used this inscribed sphere method. It avoided any influence from irregularities of the posterior eye shape in eyes with staphylomas that could have skewed the pupillary axis. The origin of the coordinate system was set 12 mm posterior to the corneal vertex, approximating the center of a normal eyeball and referred to as the hypothetical pre-elongation eye center. The posterior scleral topography was generated in a posterior region spanning 120° (Fig. 1B). For the assessment of the topography, we determined two key parameters: the distance (D) from each point to the hypothetical pre-elongation eye center, and the curvature radius (C) at each point. The distance could reveal a potential eyeball elongation or eyeball bulging if it was greater than 12 mm. The curvature was based on how well a small sphere fitted the local surface points within a radius of 3 mm (Figure 1C). From this topography, the location and extents of posterior scleral protrusion could be viewed, similar to a corneal topography.

### Development of Parameters to Characterize Posterior Scleral Morphology

Using the distance (D) and curvature (C) values, several parameters were developed to characterize the posterior scleral shape as follows:

D_mean_: The mean value of D, representing the mean posterior scleral deviation from the hypothetical pre-elongation eye center.
C_mean_: The mean value of C, representing the mean curvature of the posterior sclera within the 120° range.
D_max_: 95th percentile value of D, serving as an approximation for the maximum distance of the posterior sclera offset from the hypothetical pre-elongation eye center. We used the 95th percentile value rather than the true maximum value as a more robust estimate to avoid the potential influence of outlier points.
C_max_: 95th percentile value of C as an approximation of the maximum curvature (sharpest bend) of the posterior scleral surface. Again, the 95th percentile value was used for robustness.
D_var_: The variance of D, quantifying how far the surface deviated from a standard spherical surface. A spherical shape would have a value of zero and any deviation from a sphere result in a value larger than zero.
C · D: The product of C and D at individual points. The mean and maximum values of C · D were also calculated as C · D_mean_ and C · D_max_ using similar approaches as described above. This parameter combined the effects of scleral distance from the hypothetical pre-elongation eye center and the impact of local curvature. A point far from the hypothetical pre-elongation eye center (indicating more marked elongation or bulging) with a larger curvature (sharper protrusion) would be worse as the distance value is amplified by the curvature value.

### Qualitative Classification of Staphyloma Types

**Figure 2.**
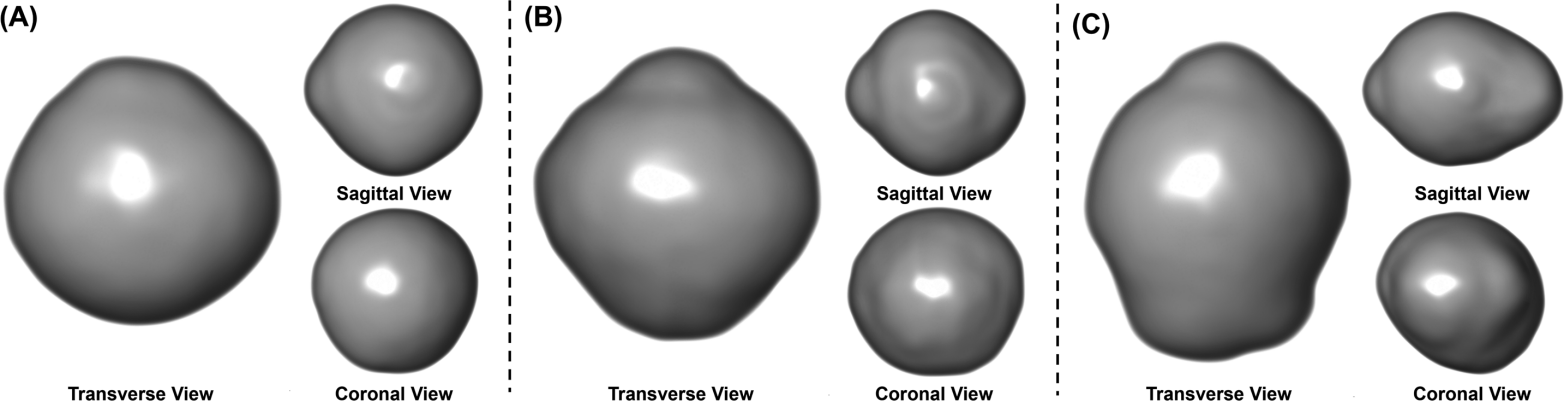
Three categories of eye shapes with three different views of the eyeball reconstructed from MRI images. This visualization was based on the segmented eyeball labels and was performed using the Volume Rendering module in the Amira software, using advanced features such as setting lighting to Specular and shade effects to Ambient. (**A**) Type 0 without apparent posterior globe protrusion. (**B**) Type 1 with elongated posterior and conical protrusion. (**C**) Type 2 with elongated posterior and sharp nasal or temporal protrusion.

To assess how well our developed parameters correlated with conventional staphyloma classifications, we manually classified the staphyloma type according to the method described by Guo et al.^14^ This classification was performed by two experienced graders using 3D MRI and ultrasound images. Three distinct categories of eye shape were defined (Fig. 2):

Type 0: Nasal and temporal symmetry with elongated posterior globe without apparent posterior globe protrusion.
Type 1: Nasal and temporal symmetry with elongated posterior and conical protrusion.
Type 2: Nasal or temporal protrusion with elongated posterior globe.

### Assessing Retinal Complications Associated With Staphyloma and OCT Imaging

Each eye's macular region was imaged with spectral-domain OCT (Spectralis; Heidelberg Engineering GmbH, Heidelberg, Germany) before and after the MRI scan. Each OCT raster scan comprised 31 horizontal B-scans (each composed of 768 A-scans) covering a rectangular region of 10.0 ± 1.1 mm (width) × 7.5 ± 1.1 mm (height) centered on the macula. The distance between consecutive B-scans varied slightly across eyes and averaged 278.7 ± 27.2 µm. Similarly, the lateral resolution of each B-scan varied slightly across eyes and averaged 13.1 ± 1.3 µm horizontally. The axial resolution was fixed at 3.9 µm. Each B-scan was averaged 20 times during acquisition to reduce speckle noise.

The correlation of the developed parameters with myopic macula changes was examined using the myopic traction maculopathy (MTM) grades based on the ATN classifications described by Ruiz-Medrano et al.,^23^ which included atrophic (A), tractional (T), and neovascular (N) changes. MTM is typically classified into six stages: T0, no myopic schisis; T1, inner or outer foveoschisis; T2, inner and outer foveoschisis; T3, foveal detachment; T4, full-thickness macular hole; T5, full-thickness macular hole and retinal detachment. Due to limited data in this study, we categorized them into 3 groups: T0, T1-2 and T3-5. All MTM diagnoses were made and confirmed by macular OCT scans and corresponding fundus images. The correlation between the well-performing parameters and MTM grades was evaluated using Lasso regression to adjust for age and AL.

### Statistical Analysis

We described the parameters by their means ± standard deviations. We used generalized estimating equations to determine the statistical significance of differences between groups, accounting for intereye correlation. *P* values adjusted by Bonferroni´s correction were considered statistically significant if less than 0.05. The area under the receiver operating characteristic curve (AUC) was used and to assess the performance of parameters in classifying the various staphyloma types. Additionally, partial AUC at specificity of 85% to 100%^24^^,^^25^ was also calculated. The statistical analysis was performed using a statistical software program (Python 3.11, with packages of Pingouin 0.5, Pandas 2.0, and NumPy 1.25).

## Results

### Subjects, MRI Images, and Automatic Segmentation Model

The study included 102 eyes from 52 subjects with a mean age of 60.570 ± 13.399 years and a mean axial length of 28.482 ± 2.355 mm. The three staphyloma type groups differed significantly in axial length (all *P* < 0.001) (Table 1).

**Table 1.** Demographic Characteristics and Morphological Parameters of Various Staphyloma Categories

	Mean ± SD			*P* Value (Adjusted)			AUC		
Parameters	Type 0 (n = 39)	Type 1 (n = 53)	Type 2 (n = 10)	Type 0 vs. Type 1	Type 0 vs. Type 2	Type 1 vs. Type 2	Type 0 vs. Type 1	Type 0 vs. Type 2	Type 1 vs. Type 2
Age (year)	53.158 ± 16.728	65.462 ± 7.280	63.300 ± 12.065	0.003	0.276	>0.999	0.725	0.668	0.380
Gender (female)	28.90%	32.70%	40.00%	>0.999	>0.999	>0.999	0.519	0.555	0.537
Axial length (mm)	27.058 ± 1.598	28.808 ± 2.101	31.978 ± 1.432	<0.001	<0.001	<0.001	0.750	**>0.999**	0.895
Spherical equivalent (D)	−7.390 ± 6.442	−10.398 ± 6.607	−4.100 ± 12.873	0.243	>0.999	0.912	0.358	0.606	0.643
C_mean_ (mm^−1^)	0.071 ± 0.005	0.068 ± 0.003	0.067 ± 0.002	0.081	0.006	0.435	0.356	0.236	0.370
C_max_ (mm^−1^)	0.101 ± 0.015	0.117 ± 0.011	0.130 ± 0.014	<0.001	<0.001	0.027	0.876	0.944	0.762
D_mean_ (mm)	16.237 ± 1.311	16.929 ± 1.314	18.699 ± 0.888	0.126	<0.001	<0.001	0.637	**0.956**	0.870
D_max_ (mm)	17.496 ± 1.586	19.352 ± 1.779	22.510 ± 1.551	<0.001	<0.001	<0.001	0.781	**>0.999**	**0.934**
D_var_ (mm²)	0.623 ± 0.512	1.795 ± 0.934	4.982 ± 1.834	<0.001	<0.001	<0.001	0.875	**>0.999**	**0.981**
C · D_mean_ (Dimensionless)	1.146 ± 0.054	1.170 ± 0.065	1.281 ± 0.036	0.243	<0.001	<0.001	0.617	**0.995**	**0.957**
C · D_max_ (Dimensionless)	1.712 ± 0.269	2.206 ± 0.260	2.833 ± 0.471	<0.001	<0.001	<0.001	**0.923**	**0.982**	**0.911**

*P*-values in bold are statistically significant. AUC values in bold are greater than 0.9.

The automated segmentation model achieved an average Dice coefficient of 0.935 for the eyeballs in the test dataset, with a 95% confidence interval from 0.917 to 0.953, a metric deemed excellent in the realm of medical image segmentation. Comprehensive details regarding the deep learning model's performance are presented in the supplementary material.

### Posterior Scleral Topography

**Figure 3.**
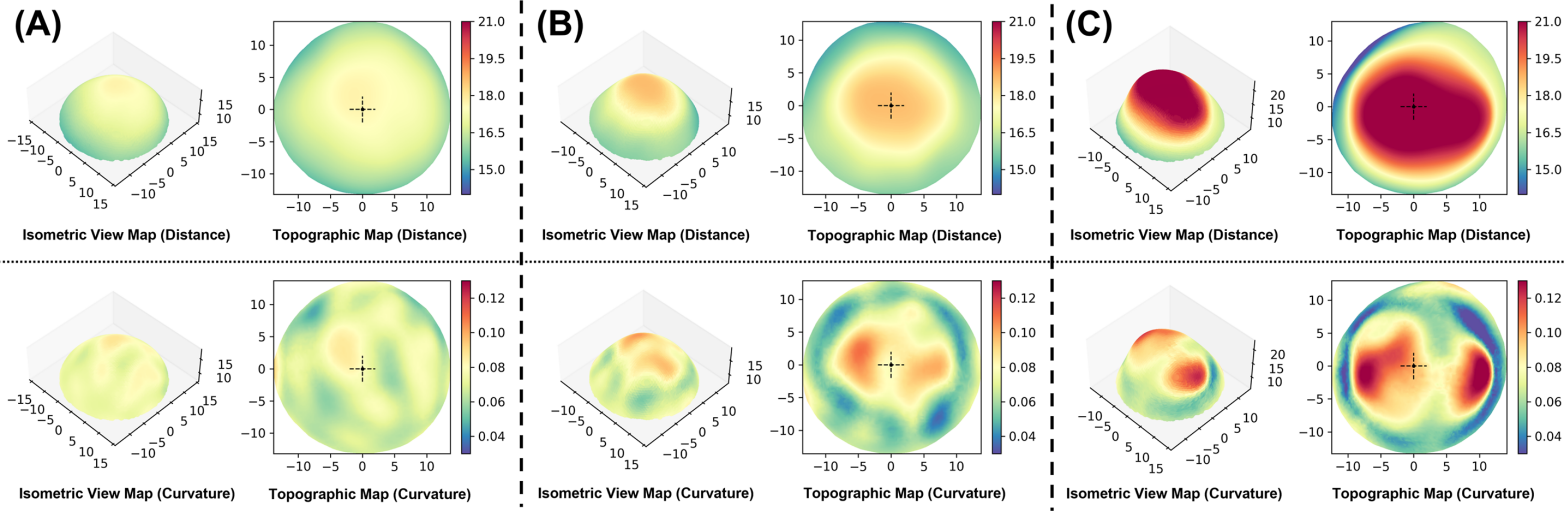
Posterior scleral topography map with different eye shapes. (**A**) Type 0 without apparent posterior globe protrusion. (**B**) Type 1 with protrusion of the center section. Distance (*D*) and curvature (*C*) in the center of the posterior surface were significantly higher than the surrounding area and the bulging was already clearly visible. (**C**) Type 2 with a much larger protrusion. *D* and *C* were overall significantly higher overall and the bulging was more pronounced on both the temporal and nasal sides.

The automatically generated posterior scleral topography map showed extent and location of scleral bulging and irregularities in the curvature distribution of the staphyloma (Fig 3).

### Distribution of Scleral Morphology Parameters

Table 1 displays the distribution of each developed parameter across the manually classified staphyloma types, with most parameters revealing variations among the different staphyloma types.

### Performance of Morphology Parameters in Conventional Classification of Staphyloma

**Figure 4.**
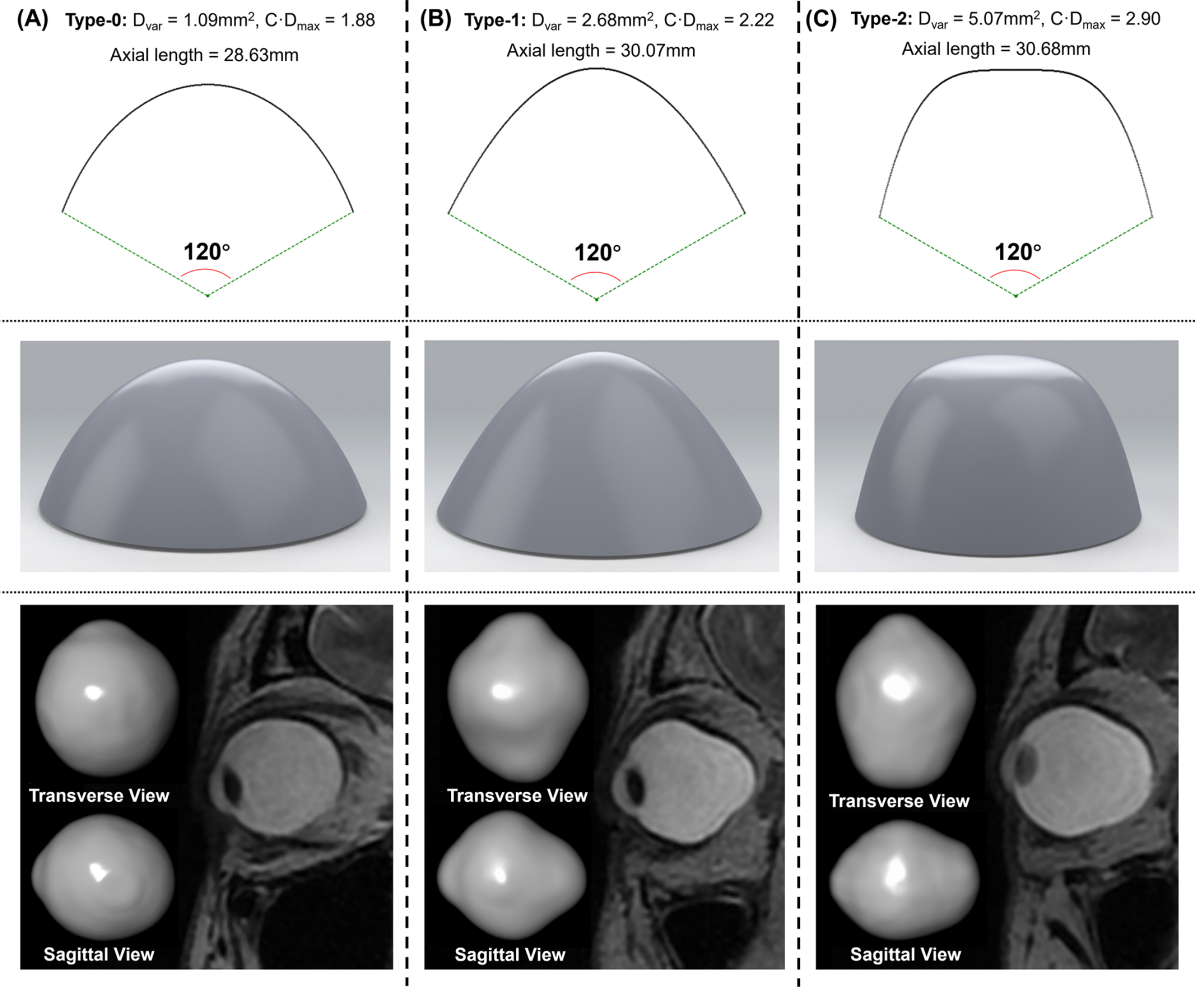
Different C · D_max_, D_var_, and AL corresponding to three categories of eye shapes. The images in the *first panel* illustrated curves drawn according to the ideal values of D_var_, spanning a 120° range. In the *second panel*, the 3D diagram represented the ideal 3D surface shape that corresponded to the assigned parameters. In the *third panel*, the MRI image depicted the parameters in the sagittal plane of the eyeball respectively. (**A**) Type 0 without apparent posterior globe protrusion. (**B**) Type 1 with protrusion of the center section. D_var_ and C · D_max_ were larger than in (**A**) because of larger elongation and local protrusion of the posterior eyeball. (**C**) Type 2 with a much larger protrusion. More severe overall elongation and protrusion of the eyeball resulted in a much more D_var_ and C D_max,_ although the axial length of this eye was similar to (**B**).

The parameter that most effectively differentiated between Type 0 and Type 1 staphyloma was C · D_max_, with an AUC value of 0.923 (Table 1). D_var_ was the most efficient at distinguishing between Type 1 and Type 2 staphyloma, although D_max_, C · D_mean_, and C · D_max_ also yielded strong results in this differentiation. Given that separating Type 0 from Type 2 staphyloma was relatively straightforward, numerous morphological parameters, including axial length, demonstrated good performance. For the comprehensive discrimination among the three staphyloma types, C · D_max_ exhibited the best overall efficacy, with all AUC values exceeding 0.9 (Fig. 4).

Additionally, the best partial AUC values (normalized by dividing by 0.15) achieved for differentiating between Type 0 and Type 1 staphyloma and Type 0 and Type 2 staphyloma were 0.614 (C · D_max_) and 0.872 (D_var_), respectively (Supplementary Fig. S4 and Supplementary Table S2 in the Supplementary Material). Type 1 and Type 2 can be well differentiated by numerous morphological parameters, including axial length and D_var_.

### Correlation of Morphologic Parameters With Retinal Complications

In our study, a total of 85 eyes from 44 individuals were used for MTM grading. Among these, 38 eyes had no MTM (T0), whereas 47 eyes exhibited MTM lesions. Of the eyes with MTM, 32 had mild to moderate MTM (T1-2) and 15 had severe MTM (T3-5).

**Figure 5.**
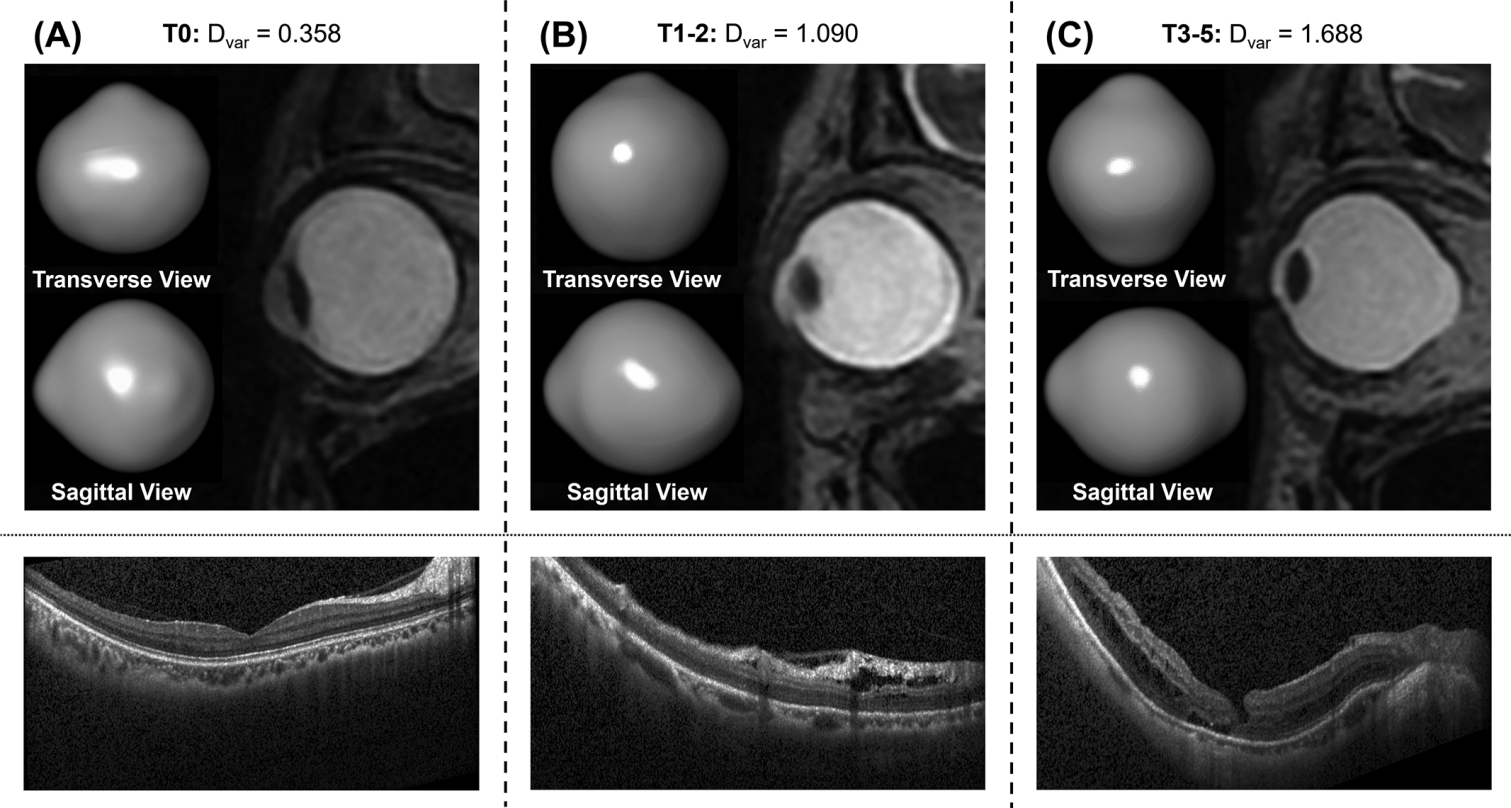
Different D_var_ and images for three MTM categories based on the ATN classifications. (**A**) T0 with a low D_var._ No MTM was observed in the retinal layer of the macular area. (**B**) T1-2 with lightly larger D_var_ and retinoschisis. (**C**) T3-5 with further larger D_var_, retinoschisis, and macular hole.

After adjusting for age and axial length, significant correlations were found between D_var_ and C · D_max_ with the grades of MTM based on the ATN classifications (Table 2). Specifically, the beta coefficients were 0.752 mm^−^^2^ (*P* = 0.022) for D_var_ and 2.435 (*P* = 0.016) for C · D_max_ (Figure 5). In the context of Lasso regression analysis, the beta coefficient represents the magnitude of change in the MTM grade for a one-unit change in the predictor variable, assuming all other variables remain constant.

**Table 2.** Correlation Between Disease Categories of MTM Grades Based on ATN Classifications and Measured Parameters

	Mean ± SD					
Parameters	T0 (n = 38)	T1-2 (n = 32)	T3-5 (n = 15)	Spearman Coefficient	Beta Coefficient	*P* Value
C_mean_ (mm^−1^)	0.070 ± 0.004	0.068 ± 0.004	0.067 ± 0.004	−0.256	74.502	0.347
C_max_ (mm^−1^)	0.106 ± 0.016	0.117 ± 0.013	0.119 ± 0.016	0.403	33.100	0.068
D_mean_ (mm)	16.173 ± 1.307	17.207 ± 1.186	18.026 ± 1.396	0.465	0.170	0.531
D_max_ (mm)	17.558 ± 1.572	19.737 ± 1.475	21.024 ± 2.352	0.640	0.432	**0.044**
D_var_ (mm²)	0.675 ± 0.631	2.010 ± 0.827	3.145 ± 2.481	0.705	0.752	**0.022**
C · D_mean_ (Dimensionless)	1.131 ± 0.064	1.193 ± 0.043	1.229 ± 0.068	0.566	10.725	**0.048**
C · D_max_ (Dimensionless)	1.813 ± 0.300	2.231 ± 0.257	2.459 ± 0.573	0.623	2.435	**0.016**

Values in bold are statistically significant.

## Discussion

This study presents a novel approach for analyzing posterior scleral staphylomas by automatically generating a posterior sclera topography from 3D MRI images. The topography allows for a visualization and quantification of staphyloma location and severity, overcoming the limitations of subjective assessments used in previous studies relying on 3D MRI. Quantitative parameters derived from the topography data, particularly D_var_ and C · D_max_, demonstrated a statistically strong agreement with conventional classification methods for staphylomas. Moreover, these two parameters correlated well with the grades of MTM based on ATN classifications, suggesting their potential correlation with the disease progression.

Although prior research used 3D MRI for analyzing and classifying posterior scleral staphyloma, the studies predominantly depended on subjective evaluations of the ocular shape. Such a methodology is, however, intrinsically subjective, constraining both repeatability and scalability. Conversely, the present study automated the assessment of the posterior scleral topography and introduced parameters to quantify shape, thus rendering the assessment of posterior staphyloma more objective and standardized.

Among the parameters developed, D_var_ and C · D_max_ demonstrated significant capabilities in differentiating various types of posterior staphyloma, which were manually classified using conventional subjective methods. D_max_ was effective in distinguishing Type 2 from Type 0 and Type 1, yet its performance in differentiating between Type 0 and Type 1 was comparatively weaker. In pairwise comparisons across the three staphyloma types, C · D_max_ consistently achieved AUC values greater than 0.9, particularly excelling in distinguishing Type 0 from Type 1. Because of the clear distinction between Type 0 and Type 2, seven parameters, including axial length, yielded good results in the differentiation. Our findings aligned well with the conventional method, highlighting our method's validity. Furthermore, the partial AUCs at a specificity of 85% to 100%, which was reported to be clinically relevant,^24^^,^^25^ were highest for D_var_ and C · D_max,_ consistent with their overall AUC performance. It is important to recognize, however, that the conventional method is subjective and should not be regarded as the definitive standard. Our research established a foundation for quantifying posterior scleral shape in future studies and clinical assessments.

The calculations of D_var_ and C · D_max_ were anchored on comparing the posterior scleral surface to a standard sphere with a radius of 12 mm. D_var_ quantified the deviation from this sphere, illustrating the extent of asphericity or distortion. Conversely, C · D_max_ encapsulated the scleral distance from the hypothetical pre-elongation eye center, indicative of elongation or bulging, and the local curvature, reflecting distortion. Figure 4 shows the D_var_ and C · D_max_ values across the three staphyloma types. For an eyeball with an axial length of 24 mm and without posterior staphyloma, D_var_ would tend toward 0, whereas C · D_max_ would be close to 1. In Figure 4A, the eye exhibited elongation, leading to a C · D_max_ > 1, yet the posterior surface remained spherical, keeping D_var_ near 1. In the case of a sharper protrusion, as seen in Figure 4B, D_var_ was comparatively higher. Figure 4C depicted an eye that was more elongated with a rougher posterior scleral surface, resulting in elevated values for both D_var_ and C·D_max_. Integrating these parameters with the generated topography offered a detailed and intuitive evaluation of the posterior scleral shape and staphyloma severity.

The clinical relevance of our approach was underscored by the established link between the posterior staphyloma shape and various characteristics of myopic macular degeneration.^20^^,^^23^^,^^26^^,^^27^ It was shown by the correlation between D_var_ and C·D_max_ and the grades of MTM based on ATN classifications.

The topography and parameters we developed were based on 3D MRI images. Currently, 3D MRI is not routinely performed in clinical ophthalmology. However, these parameters can be adapted for OCT images. Traditional OCT imaging has a limited field of view, restricting the assessment to a relatively small posterior region. It may potentially lead to missing posterior scleral staphylomas. However, with advancements in OCT technology, commercial ultra-widefield OCT systems have become increasingly available, supporting extensive scan ranges of up to 23 mm in width with a depth exceeding 20 mm.^28^^–^^31^ These capabilities enable the capture and segmentation of the posterior scleral shape, with deep learning techniques aiding in this process. It is crucial to acknowledge that OCT imaging may be influenced by optical distortions. Although advanced OCT systems include software algorithms to correct for recognized sources of optical distortion, the precision of these corrections in ultrawide field OCT, particularly for aspherically-shaped eyes with staphyloma, necessitates additional validation. Future research will involve comparing measurements from MRI and wide-field OCT in eyes of different shapes to ascertain the concordance between these two modalities and to validate the applicability of the parameters, found in the present study, with OCT images.

In conclusion, this study established a novel and objective method for quantifying posterior scleral staphylomas using 3D MRI-derived topography and associated parameters. These parameters demonstrated a strong agreement with conventional classification methods and offer potential for improved assessment of staphyloma severity. Future research focusing on adapting these parameters to wide-field OCT imaging may facilitate broader clinical application and enhance understanding of the relationship between posterior staphyloma and associated retinal pathologies.

## Supplementary Material

Supplement 1
