# Supplementary material for: Automated Posterior Scleral Topography Assessment for Enhanced Staphyloma Visualization and Quantification With Improved Maculopathy Correlation
Source: Transl Vis Sci Technol. 2024 Oct 30;13(10):41. doi: 10.1167/tvst.13.10.41 (PMC11534019; doi:10.1167/tvst.13.10.41)
Supplement: Supplement 1 [file tvst-13-10-41_s001.docx]

**Supplementary Materials**

**1. Deep Learning Model Architecture**

This study employed deep learning for the segmentation of the eyeball from MRI images. **Figure S1** illustrates the architecture of the neural network utilized for this task. The network's design is further detailed within the figure, specifically highlighting the feature block. To demonstrate the effectiveness of the approach**, Figure S2** presents the results for three typical images. These comparisons visually depict the segmentation achieved through both manual and automated methods.


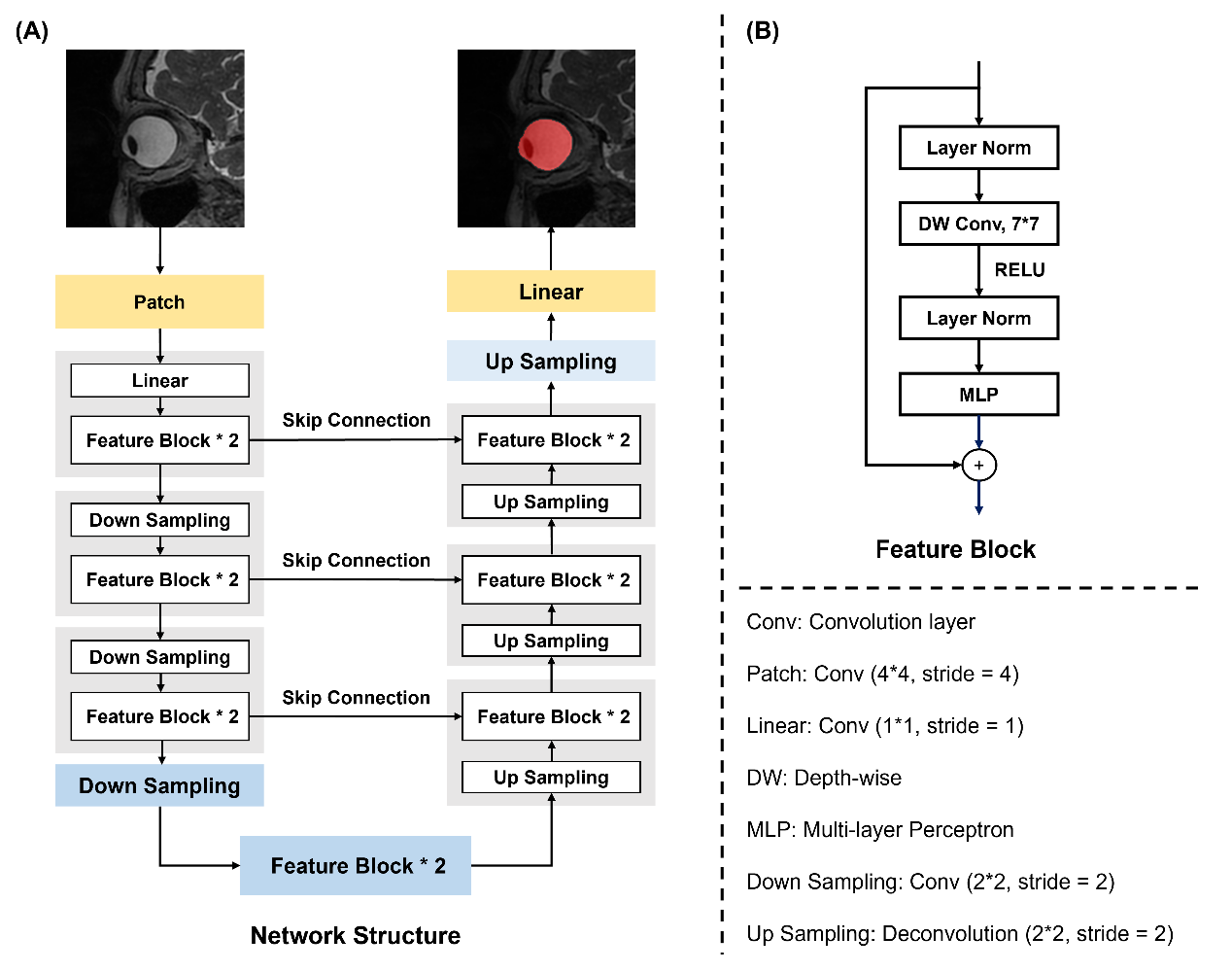


**Figure S1.** The deep neural-network architecture for automated segmentation of the eyeball from MRI images. (**A**) The whole network structure. (**B**) Details of the feature block of the network.


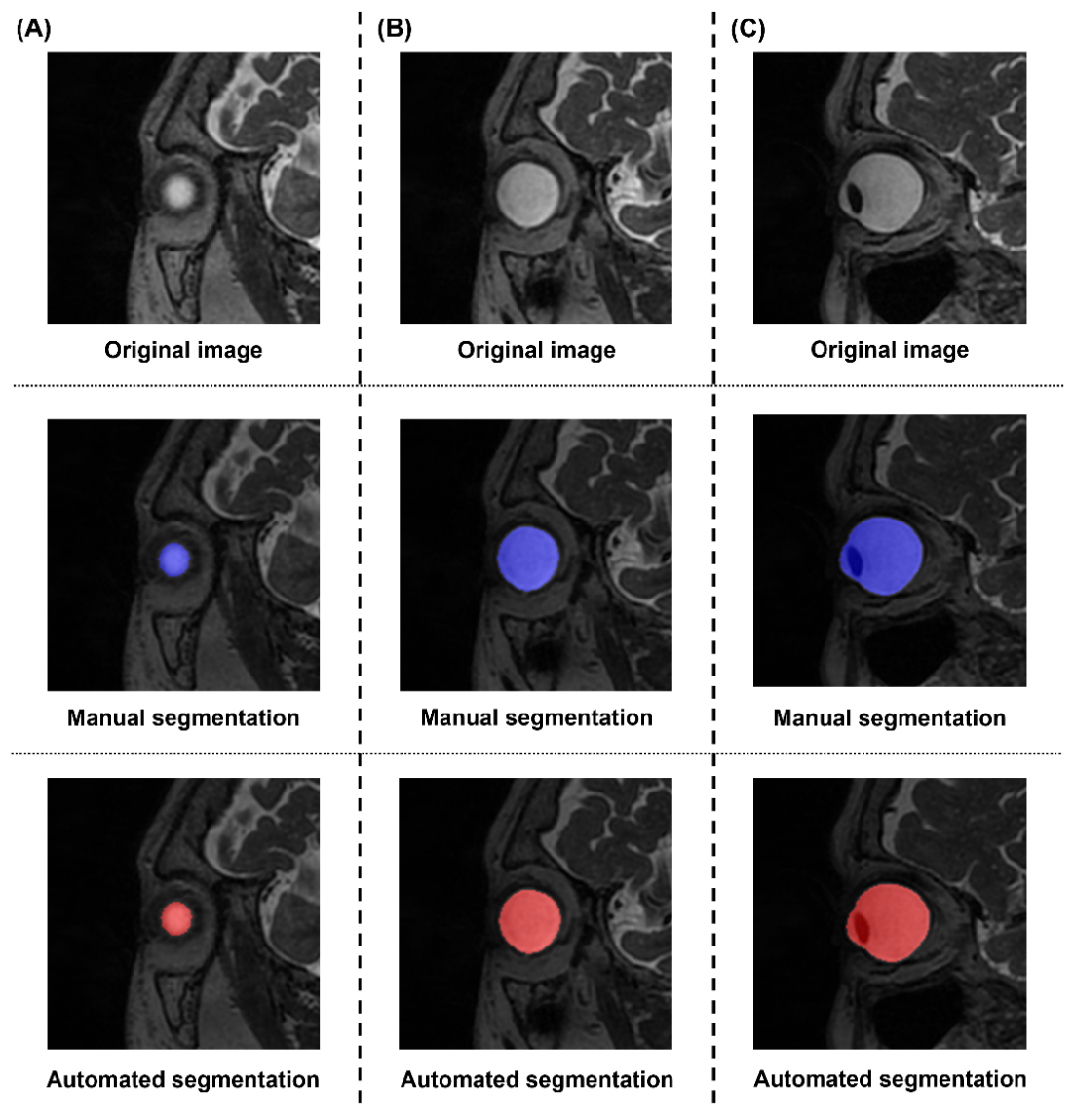


**Figure S2.** Slices of the eyeball at different sagittal positions. Each subfigure shows the same slice: (**A**) original image, (**B**) manual segmentation, and (**C**) automated segmentation.

**2. Validity of the Methods to Determine Pupillary-Foveal Axis**

The validity of our method for determining the pupillary-foveal axis was assessed by comparing it with the method proposed by Hoang et al. (*British Journal of Ophthalmology*, 105.8 (2021): 1149-1154). Using the method of Hoang et al., the limbal points on each 2D slice from 20 randomly selected eyes were manually marked to fit a limbal plane and determine its normal vector. The average angle difference between our method and Hoang et al. was 4.24 ± 1.94°, with a 95% confidence interval of 3.33 to 5.15°. **Figure S3** and **Table S1** show good agreement in posterior scleral morphological parameters calculated based on the pupil-foveal axis obtained from both methods.


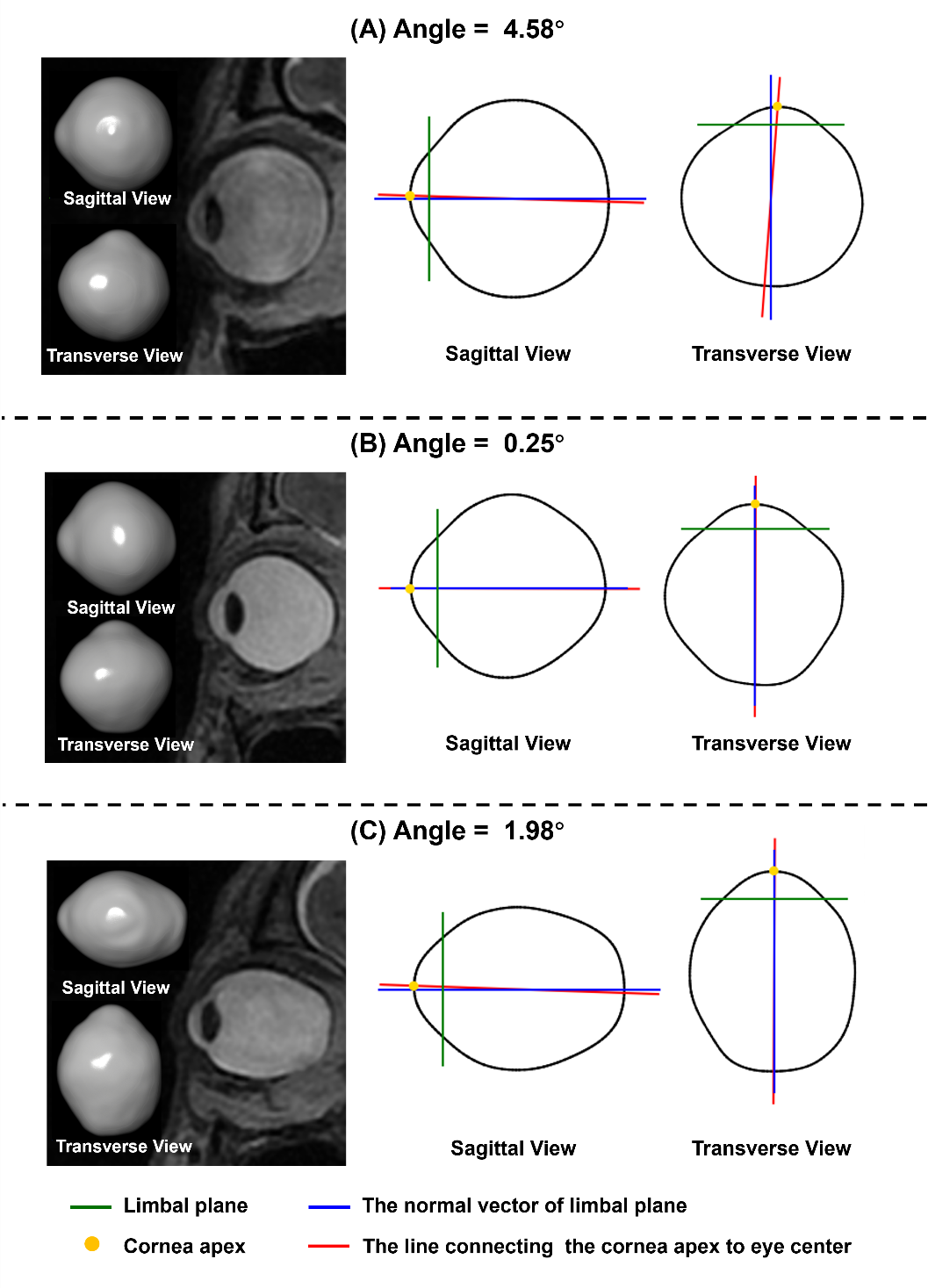


**Figure S3.** Comparison of pupillary-foveal axis obtained using our method (red line) and Hoang et al.'s method (blue line) for three categories of eye shapes. (A) Type-0. (B) Type-1. (C) Type-2.

| **Table S1. Comparison of Morphology Parameters Calculated Based on the Pupillary-Foveal Axis Obtained from the Method Proposed by Hoang et al. and Our Method.** | | | | |
| --- | --- | --- | --- | --- |
| **Parameters** | **Mean ± Std  (Hoang et al. method)** | **Mean ± Std  (Our method)** | **P Value** | **ICC** |
| **C_mean_ (mm^-1^)** | 0.069 ± 0.003 | 0.068 ± 0.003 | 0.177 | 0.959 |
| **C_max_ (mm^-1^)** | 0.107 ± 0.018 | 0.113 ± 0.020 | 0.277 | 0.988 |
| **D_mean_ (mm)** | 17.034 ± 1.579 | 17.286 ± 1.566 | 0.616 | 0.997 |
| **D_max_ (mm)** | 19.317 ± 2.535 | 19.737 ± 2.553 | 0.605 | 0.994 |
| **D_var_ (mm²)** | 2.140 ± 1.952 | 2.326 ± 2.175 | 0.778 | 0.988 |
| **C·D_mean_ (Dimensionless)** | 1.190 ± 0.078 | 1.185 ± 0.081 | 0.842 | 0.988 |
| **C·D_max_ (Dimensionless)** | 2.031 ± 0.558 | 2.199 ± 0.618 | 0.373 | 0.989 |
| ICC = inter-class correlation coefficients. | | | | |

**3. Partial AUC Results**

To enhance the evaluation of how well the measured parameters differentiate between various Staphyloma categories, the partial area under the receiver operating characteristic curve (AUC) was computed within the 85–100% specificity range, which would be clinically relevant for screening. **Figure S4** and **Table S2** illustrate the effectiveness of different measurement parameters in distinguishing between various Staphyloma categories. Partial AUCs were normalized by dividing by 0.15.


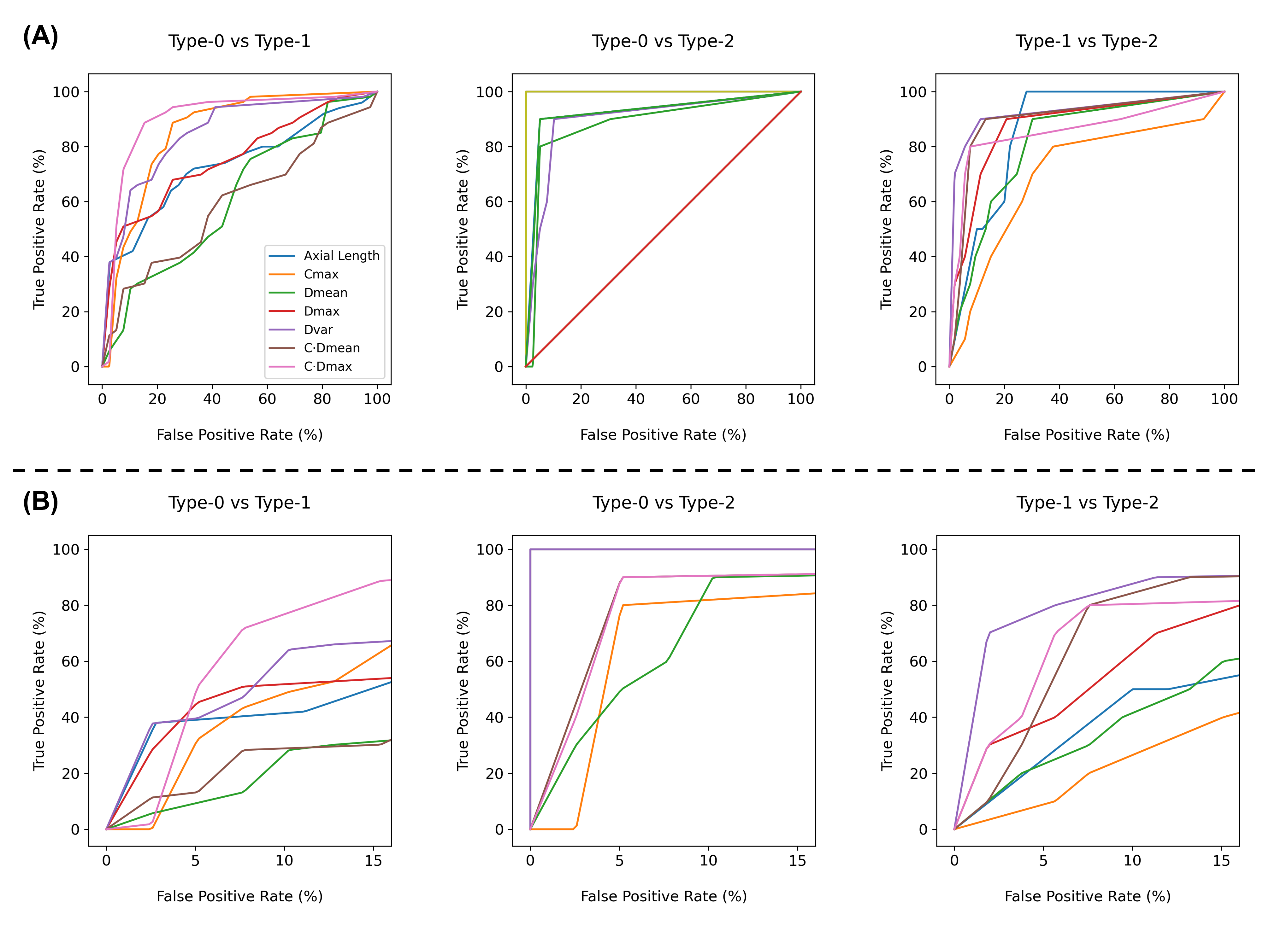


**Figure S4.** The AUC **(A)** and partial AUC **(B)** of different parameters for the differentiation of different staphyloma categories. Age, gender, and **C_mean_** are not included in the figure due to their poor performance (low AUC).

| **Table S2. AUC and Partial AUC for Morphological Parameters of Various Staphyloma Categories.** | | | | | | |
| --- | --- | --- | --- | --- | --- | --- |
| **Parameters** | **Type-0 vs Type-1** | | **Type-0 vs Type-2** | | **Type-1 vs Type-2** | |
|  | **AUC** | **Partial AUC** | **AUC** | **Partial AUC** | **AUC** | **Partial AUC** |
| **Age (year)** | 0.725 | <0.100 | 0.668 | 0.200 | 0.380 | 0.200 |
| **Gender (famale)** | 0.519 | <0.100 | 0.555 | 0.104 | 0.537 | <0.100 |
| **Axial Length (mm)** | 0.750 | 0.433 | **>0.999** | **>0.999** | 0.895 | 0.457 |
| **Spherical Equivalent (Dioptre)** | 0.358 | <0.100 | 0.606 | 0.411 | 0.643 | 0.478 |
| **C_mean_ (mm^-1^)** | 0.356 | <0.100 | 0.236 | <0.100 | 0.370 | <0.100 |
| **C_max_ (mm^-1^)** | 0.876 | 0.410 | **0.944** | 0.728 | 0.762 | 0.261 |
| **D_mean_ (mm)** | 0.637 | 0.204 | **0.956** | 0.706 | 0.87 | 0.361 |
| **D_max_ (mm)** | 0.781 | 0.478 | **>0.999** | **>0.999** | **0.934** | 0.600 |
| **D_var_ (mm²)** | 0.875 | 0.532 | **>0.999** | **>0.999** | **0.981** | **0.872** |
| **C·D_mean_ (Dimensionless)** | 0.617 | 0.231 | **0.995** | **0.961** | **0.957** | 0.694 |
| **C·D_max_ (Dimensionless)** | **0.923** | **0.614** | **0.982** | 0.878 | **0.911** | 0.711 |
| AUC = The area under the receiver operating characteristic curve. | | | | | | |
